# Protocol to study *in vivo* organ-specific migration of apoptotic splenocytes in mice with tumor and immune checkpoint inhibitor-induced colitis

**DOI:** 10.1016/j.xpro.2025.103878

**Published:** 2025-06-05

**Authors:** Lukas M. Braun, Robert Zeiser

**Affiliations:** 1 Department of Internal Medicine I, Medical Center – University of Freiburg, Faculty of Medicine, University of Freiburg, Freiburg, Germany; 2 German Cancer Consortium (DKTK), Partner Site Freiburg, a partnership between DKFZ and Medical Center – University of Freiburg, Freiburg, Germany; 3 Signalling Research Centres BIOSS and CIBSS – Centre for Integrative Biological Signalling Studies, University of Freiburg, Freiburg, Germany

**Keywords:** cancer, immunology, model organisms

## Abstract

Extracorporeal photopheresis (ECP) reduces immune checkpoint inhibitor (ICI)-induced colitis without affecting anti-tumor immunity. Here, we provide a protocol to trace ECP-treated cells *in vivo* after transfer into tumor-bearing mice with ICI-induced colitis. We detail tumor and colitis induction, ICI therapy, and the labeling of ECP-treated splenocytes to trace their migration into target organs. Fluorescence imaging and tissue digestion enable the analysis of phagocytic uptake and the analysis of tolerogenic polarization of phagocytes based on congenic markers of donor and recipient mice.

For complete details on the use and execution of this protocol, please refer to Braun et al.^1^

## Highlights

• Study *in vivo* migration of fluorescently labeled cells into different target organs
• Instructions for investigating phagocytic uptake of cells *in vivo*
• Guidance on inducing cell death with extracorporeal photopheresis
• Procedures for isolating immune cells from lamina propria and tumor tissue

## Abstract (editor highlights)

Publisher’s note: Undertaking any experimental protocol requires adherence to local institutional guidelines for laboratory safety and ethics.

## Graphical abstract

**Figure:**
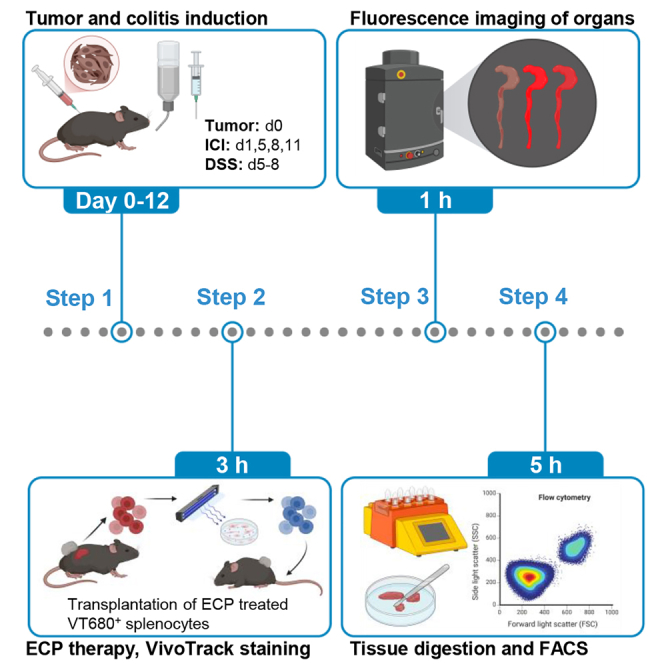

## Before you begin

This protocol describes the specific steps to identify *in vivo* migration and phagocytic uptake of splenocytes treated with Extracorporeal Photopheresis (ECP) in C57BL/6 mice transplanted with s.c. melanoma and immune checkpoint inhibitor (ICI)-induced colitis. ECP treatment renders splenocytes apoptotic. We use male and female C57BL/6N (CD45.2, Janvier Labs) and CD45.1 (Charles River) mice with an age of 7–10 weeks in the following protocol. We transplant recipient (CD45.2) and donor (CD45.1) mice subcutaneously with syngeneic B16.F10 melanoma cells and treat them with Dextran Sodium Sulfate (DSS, colitis grade) and anti-PD-1 to induce ICI-associated colitis. Colitis following ICI is one of the major limitations of ICI in the context of cancer therapy. Anti-CTLA-4 could be used as an alternative ICI, and tumor induction could also be achieved using other established syngeneic murine tumor cell lines. Previously, we have established ECP as an immunomodulatory therapy for ICI-induced colitis in mice and humans. ECP describes the treatment of immune cells with 8-methoxypsoralen and UVA, thereby inducing apoptosis in these cells. In our preclinical model, we have established splenocytes as the immune cell source for ECP therapy (see Braun et al., 2025^1^). To discriminate recipient and donor immune cells in organ analysis, we transfer splenocytes from CD45.1 donor mice into CD45.2 recipient mice. The donor and recipient mice are treated similarly; this includes transplantation with syngeneic tumor, and treatment with DSS and anti-PD-1. ECP-treated splenocytes are stained with a fluorescent dye to track the migration of the cells into specific organs using an IVIS Lumina III imaging system. It is important to apply a chlorophyll-free diet to the mice for ∼14 days before the imaging experiment to reduce the background fluorescence.

### Institutional permissions

Any experiments on live vertebrates or higher invertebrates must be performed in accordance with relevant institutional and national guidelines and regulations. Animal studies were carried out in compliance with relevant animal-use guidelines and ethical regulations. All protocols were approved by the Regierungspräsidium Freiburg, Germany (Protocol approval number: G-20/160).

C57BL/6 mice were purchased either from Janvier Labs (France) or obtained from the local stock of the animal facility at University Medical Center Freiburg (Germany). B6.SJL-*Ptprc*^*a*^*Pepc*^*b*^/BoyCrl (Ly5.1, CD45.1) mice were originally purchased from Charles River. All mouse strains were bred at the local animal facility at University Medical Center Freiburg (Germany). All mice were housed under specific pathogen-free conditions at the University Medical Center Freiburg (Germany). Mice were used between 7 and 10 weeks of age, and only female or male donor/recipient pairs were used.

### Culture of B16.F10 melanoma cell line

**Timing: ∼1 week**

**CRITICAL:** The chosen cell line should not express any fluorescence to minimize the fluorescence background during organ imaging.

1. Medium preparation.
  a. B16.F10 cells are grown in DMEM medium supplemented with 10% heat-inactivated FCS and 1% Penicillin/Streptomycin. Medium containing supplements is called cDMEM.
  b. Pre-warm the cDMEM to 37°C before use.
2. Thawing B16.F10 cell line.
  a. Retrieve vial to be thawed from a liquid nitrogen tank and keep the vial in dry ice until ready to thaw.
  b. Prepare a 15 mL falcon tube containing 10 mL warm cDMEM.
  c. Quickly thaw the frozen cells in the water bath at 37°C and disinfect the vial with 70% ethanol.
  d. Transfer the cells into the tube containing warm cDMEM.
  e. Spin down the cells at 300 × *g* for 5 min.
  f. Discard the supernatant.
  g. Resuspend the cell pellet in 1 mL cDMEM by pipetting.
  h. Transfer the cells into a T75 cell culture flask in a total of 13-15 mL of cDMEM.
  i. Culture the cells at 37°C and 5% CO_2_ in a humidified atmosphere.
  j. Monitor the cells daily and passage if reaching 70% confluency.
3. Passaging B16.F10 cells.
  a. Cells need to be passaged if they reach 70% confluency.
  b. Aspirate the medium from the cells.
  c. Wash once with 10 mL 1X PBS.
  d. Add 1.5 mL 0.05% Trypsin-EDTA and incubate the cells at 37°C for 3–5 min.
  e. Check under the microscope if the cells have detached from the plate.
  f. Add 8.5 mL cDMEM, resuspend by pipetting and passage cells at a ratio of ∼1:10, depending on the confluency.

### Preparing B16.F10 melanoma cells for subcutaneous transplantation

**Timing: 20 min**

4. Passage melanoma cells one day before the planned subcutaneous injection in a way to reach 50–70% confluency on the day of injection.

### Chlorophyll-free diet

**Timing: ∼2 weeks**

5. If fluorescence signals in the gastrointestinal tract are to be measured, mice should be fed a chlorophyll-free diet (e.g., AIN-93) for ∼2 weeks prior to imaging.

**CRITICAL:** Chlorophyll in the intestinal tract of mice causes high fluorescence background during the imaging. The chosen diet should be chlorophyll-free but should otherwise be comparable to the chow the mice received before.

### Dextran sodium sulfate and ICI-induced colitis

**Timing: 3 days**

6. C57BL/6N and CD45.1 mice receive 3% DSS in drinking water for 72 h.
  a. Monitor the weight of the mice daily once DSS was applied.
  b. Re-new DSS solution after 48 h.
  c. Substitute DSS with normal drinking water after 72 h.

**CRITICAL:** The DSS used for these experiments needs to be specified as “colitis grade” to ensure colitis induction. The extent of colonic epithelial injury and ulceration depends on the DSS concentration, the mouse strain, the environment (animal facility) and the amount of DSS consumed by the animals. DSS-associated colitis is associated with diarrhea, loose stool, weight loss and rectal bleeding. Elimination criteria need to be established based on local ethical regulations and closely monitored throughout the experiment.

### Preparing tumor digestion medium

**Timing: 10 min**

7. For the preparation of the tumor digestion medium, dissolve the enzymes in cDMEM medium. Prepare the digestion medium freshly before digestion.

### Preparing medium and enzymes for lamina propria dissociation

**Timing: 30 min**

8. Reconstitute the enzymes for lamina propria according to manufacturer’s instructions (Lamina Propria Dissociation Kit, Miltenyi, #130-097-410).
9. Prepare predigestion and digestion solutions freshly before lamina propria dissociation.

**CRITICAL:** DTT is not stable for long times if diluted in the predigestion solution. Add DTT only directly before using the predigestion solution.

## Key resources table

**Table:** 

REAGENT or RESOURCE	SOURCE	IDENTIFIER
**Antibodies**		

Anti-PD-1 (clone RMP1-14)	Ichorbio	Cat# ICH1132; RRID:AB_2921498
Anti-mouse/human Arginase 1 (eFluor450; clone A1exF5; dilution 1:50)	Thermo Fisher Scientific	Cat# 48-3697-82; RRID:AB_2734837
Anti-mouse/human CD11b (FITC; clone M1/70; dilution 1:20)	BioLegend	Cat# 101206; RRID:AB_312788
Anti-mouse CD45.1 (FITC; clone A20; dilution 1:100)	BioLegend	Cat# 110706; RRID:AB_313494
Anti-mouse CD45.2 (Pacific Blue; clone 104; dilution 1:100)	BioLegend	Cat# 109820; RRID:AB_492873
Anti-mouse CD45.2 (PE; clone 104; dilution 1:100)	BioLegend	Cat# 109808; RRID:AB_313444
Anti-mouse CD16/CD32 (clone 93; dilution 1:25); used to block Fc receptors	BioLegend	Cat# 101302; RRID:AB_312801

**Chemicals, peptides, and recombinant proteins**		

8-methoxypsoralen (UVADEX)	Therakos	PZN# 01087204
ACK Lysing buffer	Thermo Fisher Scientific	Cat# A1049201
Fetal bovine serum (Fetal Constance, EU approved)	Anprotec	Cat# AC-SM-0190
DMEM high glucose	Thermo Fisher Scientific	Cat# 41966029
RPMI 1640	Thermo Fisher Scientific	Cat# 21875034
IVISense 680 Fluorescent Cell Labeling Dye (VivoTrack)	PerkinElmer / Revvity	Cat# NEV12000
Collagenase IA	Sigma	Cat# C9891
Deoxyribonuclease I from bovine pancreas (DNase I)	Sigma	Cat# DN25
Dextran sodium sulfate, colitis grade	MP Biomedicals	Cat# 0216011080
DL-Dithiothreitol	Merck	Cat# 646563-10X.5ML
EDTA (0.5 M), pH 8.0	Thermo Fisher Scientific	Cat# AM9261
HBSS w/o calcium w/o magnesium w/ sodium bicarbonate w/o Phenol red	Anprotec	Cat# AC-BS-0010
HBSS w/ calcium w/ magnesium w/ sodium bicarbonate w/o Phenol red	Anprotec	Cat# AC-BS-0013
HEPES	Thermo Fisher Scientific	Cat# 15630080
Trypsin-EDTA (0.25%)	Thermo Fisher Scientific	Cat# 25200056
Penicillin/Streptomycin	Thermo Fisher Scientific	Cat# 15140122
Percoll	Cytiva	Cat# 17089101

**Critical commercial assays**		

Cytofix/Cytoperm Fixation/Permeabilization Kit	BD Biosciences	Cat# 554714
LIVE/DEAD Fixable Aqua Dead Cell Stain Kit	Thermo Fisher Scientific	Cat# L34966
Lamina Propria Dissociation Kit	Miltenyi	Cat# 130-097-410

**Experimental models: Cell lines**		

B16.F10	Gift from Hanspeter Pircher	RRID:CVCL_0159

**Experimental models: Organisms/strains**		

Mouse: C57BL/6N 7–10 weeks, male and female	Janvier Labs	RRID:IMSR_RJ:C57BL-6NRJ
Mouse: CD45.1: B6.SJL-*Ptprc*^*a*^*Pepc*^*b*^/BoyCrl 7–10 weeks, male and female	Charles River	Strain #494; RRID:IMSR_CRL:494

**Software and algorithms**		

FlowJo v.10	BD Biosciences	N/A
FACSDiva Software v.6	BD Biosciences	N/A
GraphPad Prism	GraphPad	N/A
Living Image	Revvity	Cat# 128110

**Other**		

123count eBeads Counting Beads	ThermoFisher Scientific	Cat# 01-1234-42
Alfalfa-free diet AIN-93	ResearchDiets	Cat# D10012G
BS-02 UV irradiation chamber equipped with UVA	Opsytec Dr. Gröbel	Cat# 860902; 860820
gentleMACS C tubes	Miltenyi	Cat# 130-093-237

## Materials and equipment

**Table:** Culture medium for B16.F10 cells (cDMEM)

Reagent	Final concentration	Amount
DMEM	N/A	445 mL
FCS (heat inactivated)	10%	50 mL
Penicillin/Streptomycin (10,000 units/mL)	100 units/mL	5 mL
**Total**	**N/A**	**500 mL**

Store at 4°C for up to 1 month.

**Table:** Medium for splenocyte treatment (cRPMI)

Reagent	Final concentration	Amount
RPMI	N/A	445 mL
FCS (heat inactivated)	10%	50 mL
Penicillin/Streptomycin (10,000 units/mL)	100 units/mL	5 mL
**Total**	**N/A**	**500 mL**

Store at 4°C for up to 1 month.

**Table:** Tumor digestion medium (5 mL per tumor)

Reagent	Final concentration	Amount
cDMEM (DMEM + 10% FCS + 1% P/S)	N/A	5 mL
Collagenase IA	1 mg/mL	5 mg
DNase I	0.05 mg/mL	0.25 mg
**Total**	**N/A**	**5 mL**

Prepare freshly before each digestion.

**Table:** 100% isotonic Percoll (P100)

Reagent	Final concentration	Amount
Percoll (original stock)	90%	9 mL
10X PBS	1X	1 mL
**Total**	**N/A**	**10 mL**

Prepare freshly before use. Store at 20°C–25°C.

**Table:** 70% Percoll (P70)

Reagent	Final concentration	Amount
P100 Percoll	70% (relative to P100)	7 mL
1X PBS	30%	3 mL
**Total**	**N/A**	**10 mL**

Prepare freshly before use. Store at 20°C–25°C.

**Table:** 40% Percoll (P70)

Reagent	Final concentration	Amount
P100 Percoll	40% (relative to P100)	4 mL
RPMI (w/o supplements)	60%	6 mL
**Total**	**N/A**	**10 mL**

Prepare freshly before use. Store at 20°C–25°C.

**Table:** 30% Percoll (P70)

Reagent	Final concentration	Amount
P100 Percoll	30% (relative to P100)	3 mL
1X PBS	70%	7 mL
**Total**	**N/A**	**10 mL**

Prepare freshly before use. Store at 20°C–25°C.

**Table:** Predigestion solution for lamina propria dissociation (40 mL per sample)

Reagent	Final concentration	Amount
HBSS w/o Ca^2+^ and Mg^2+^	N/A	37.16 mL
HEPES (stock 1 M)	10 mM	0.4 mL
EDTA (stock 500 mM)	5 mM	0.4 mL
FCS	5 %	2 mL
DTT (stock 1 M)	1 mM	0.04 mL
**Total**	**N/A**	**40 mL**

Prepare freshly before digestion; DTT should be added only directly before digestion.

**Table:** Digestion solution for lamina propria dissociation (2.5 mL per sample)

Reagent	Final concentration	Amount
HBSS with Ca^2+^ and Mg^2+^	N/A	2.35 mL
HEPES (stock 1 M)	10 mM	0.025 mL
FCS	5%	0.125 mL
**Total**	**N/A**	**2.5 mL**

Prepare freshly before digestion.

**Table:** Flow cytometry staining buffer

Reagent	Final concentration	Amount
dH2O	N/A	435.5 mL
PBS (stock 10X)	1X	50 mL
EDTA (stock 0.5 M)	2.5 mM	2.5 mL
FCS	2%	10 mL
NaN3 (stock 5%)	0.02%	2 mL
**Total**	**N/A**	**500 mL**

Store at 4°C for up to 3 months.

## Step-by-step method details

### Subcutaneous injection of tumor cells and anti-PD-1 treatment

**Timing: 2 h**

This step is important for the subcutaneous transplantation of tumors into recipient mice. The localized subcutaneous tumor growth is important to determine the migration of ECP-treated splenocytes into a defined tissue.

1. Use B16.F10 melanoma cells at 50–70% confluence.
  a. Aspirate the medium from the cells.
  b. Wash once with 10 mL 1X PBS.
  c. Add 1.5 mL 0.05% Trypsin-EDTA and incubate the cells at 37°C for 3–5 min.
  d. Check under the microscope if the cells have detached from the plate.
  e. Add 8.5 mL cDMEM, resuspend by pipetting and transfer into a 50 mL tube.
  f. Add 1X PBS to 40 mL.
  g. Spin down the cells at 300 × *g* for 5 min.
  h. Wash the cells twice with 1X PBS.
  i. Resuspend the cell pellet in 1X PBS by pipetting.
  j. Count the cells using a hemocytometer.
  k. Adjust the cells to 1×10^7^ cells/mL in 1X PBS.
  l. Keep the cells on ice until injection.
2. Preparation of animals.
  a. Anesthetize mice with isoflurane gas (3.5–5% in oxygen) in an anesthesia chamber (flow rate 1.5 L/min).
  b. Shave the right flank of each mouse before the tumor cell injection.
3. Injection of tumor cells (under anesthesia).
  a. Inject 100 μL of tumor cell suspension subcutaneously, i.e., 1×10^6^ cells total.
4. Treatment with anti-PD-1.
  a. Inject anti-PD-1 i.p. on days 1, 5, 8 and 11.
  b. Dilute anti-PD-1 to 2 mg/mL in 1X PBS.
  c. Apply anti-PD-1 by i.p. injection of 100 μL (i.e., 0.2 mg per mouse).

***Note:*** The number of subcutaneously injected tumor cells is dependent on the tumor cell line. The anesthesia depends on local animal welfare regulations and needs to be applied based on local guidelines.

***Note:*** Palpable tumors should appear within 5 days after injection. B16.F10 melanoma have a black color and are easy to identify if growing subcutaneously.

### Treatment with dextran sodium sulfate

**Timing: 30 min**

This section describes the application of DSS to the mice in drinking water to induce colitis. This step is critical to establish a mouse model with colitis in tumor-bearing mice to investigate organ-specific migration of ECP-treated splenocytes. DSS is given from day 5 to day 8 (72 h).

5. Replace normal drinking water with water supplemented with 3% DSS (colitis grade).
  a. Dissolve DSS in drinking water.
  b. Replace the DSS with freshly dissolved DSS in drinking water after 48 h.
  c. Replace the DSS with normal drinking water after 72 h.

***Note:*** Diarrhea and weight loss are signs for colitis development. Monitor the bodyweight of the mice to ensure colitis development.

### ECP treatment, VivoTrack680 staining, and transplantation of splenocytes

**Timing: 3 h**

**Figure 1:**
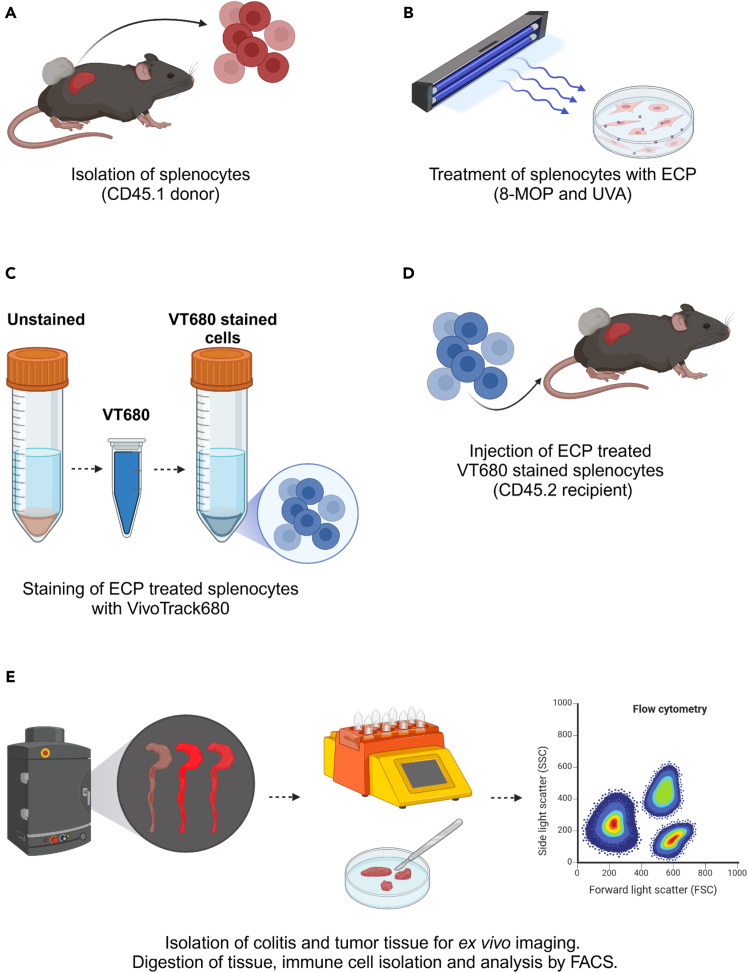
Graphical summary of *in vivo* migration analysis of ECP treated and fluorescently labeled splenocytes (A) Splenocytes are isolated from CD45.1 donor mice, which were transplanted with tumor and treated with DSS and ICI to induce colitis. (B) Subsequently, splenocytes are treated with 8-MOP and UVA, which is ECP. (C and D) The splenocytes are (C) stained with the VivoTrack680 dye and (D) intravenously transplanted into CD45.2 recipient mice. Recipients were transplanted with tumor and treated with DSS and ICI for colitis induction. (E) At defined time points after transplantation (2, 6, and 12 h), tumor and colitis tissue are isolated, dissociated and immune cells are analyzed to quantify the infiltration of ECP treated cells into these organs.

This step describes the major part of this protocol. It describes the treatment of donor splenocytes with Extracorporeal Photopheresis, the staining with VivoTrack680 to facilitate splenocyte tracking *in vivo* and the intravenous transplantation of these splenocytes into recipient animals. Splenocytes are transplanted on day 12 of the experiment. Figure 1 summarizes the experimental protocol from splenocyte isolation until tissue dissociation and analysis of immune cells by flow cytometry.

6. Treatment of splenocytes with Extracorporeal Photopheresis.
  a. Euthanize donor mice (CD45.1) by cervical dislocation or a method approved by the local animal ethics committee.
  b. Isolate the spleen.
  c. Mash the spleen through a 100 μm strainer into a 6 cm dish filled with 5 mL 1X PBS.
  d. Spin down the cells at 300 × *g* for 5 min at 4°C.
  e. Resuspend cells in 2 mL erythrocyte lysis buffer of your choice (e.g., ACK).
    i. Incubate at 20°C–25°C for 2 min.
    ii. Add 40 mL 1X PBS and spin down the cells at 300 × *g* for 5 min at 4°C.
  f. Resuspend splenocytes in cRPMI to 5×10^6^ cells/mL.
  g. Plate 12 mL cell suspension per dish into 10 cm plates.
  h. Add 8-methoxypsoralen (UVADEX) to a final concentration of 200 ng/mL.
  i. Incubate the cells for 30 min at 37°C in the dark.
    i. Apply UVA at a defined dose of 2.0 J/cm^2^ to the cells.
    ii. The BS-02 UVA chamber (Opsytec) equipped with a UV-MAT can be used in dose-controlled mode.
  j. Wash the cells twice with 1X PBS.
  k. Discard the supernatant and resuspend the cells (up to 2.5×10^8^ cells/mL) in 2 mL 1X PBS in a 50 mL tube.
7. Label cells with VivoTrack680.
  a. Dissolve one vial of VivoTrack680 in 1.3 mL warm 1X PBS (37°C) and vortex until completely dissolved; this will yield 2 mL of the labeling solution.
  b. Add 2 mL of the cell labeling solution to 2 mL of cells; mix immediately by vortexing.
  c. Incubate the cells for 15 min at 20°C–25°C in the dark.
  d. Wash the cells by adding 20 mL 1X PBS (20°C–25°C) containing 1% FCS or cRPMI.
  e. Wash the cells 2 times with 1X PBS (20°C–25°C) containing 1% FCS.
  f. Wash the cells 2 times with 1X PBS (20°C–25°C).
8. Transplant fluorescently labeled splenocytes.
  a. Resuspend cells in 1X PBS to 5×10^7^ cells/mL.
  b. Transplant mice intravenously with 200 μL cell suspension via tail vein injection (i.e., 1×10^7^ cells).

**CRITICAL:** In order to have enough VT680-stained ECP-treated splenocytes for transplantation, one donor mouse should be calculated for two recipient mice.

**CRITICAL:** It is important that the splenocytes are treated with 8-methoxypsoralen and UVA in combination to induce cell death. Either of these alone does not induce cell death in splenocytes.

***Note:*** Machines for UVA application should be calibrated regularly if not operated in a dose-controlled mode to ensure application of the correct dosage of UVA to the splenocytes.

### Fluorescence imaging of organs *ex vivo* and image analysis

**Timing: 1 h**

**Figure 2:**
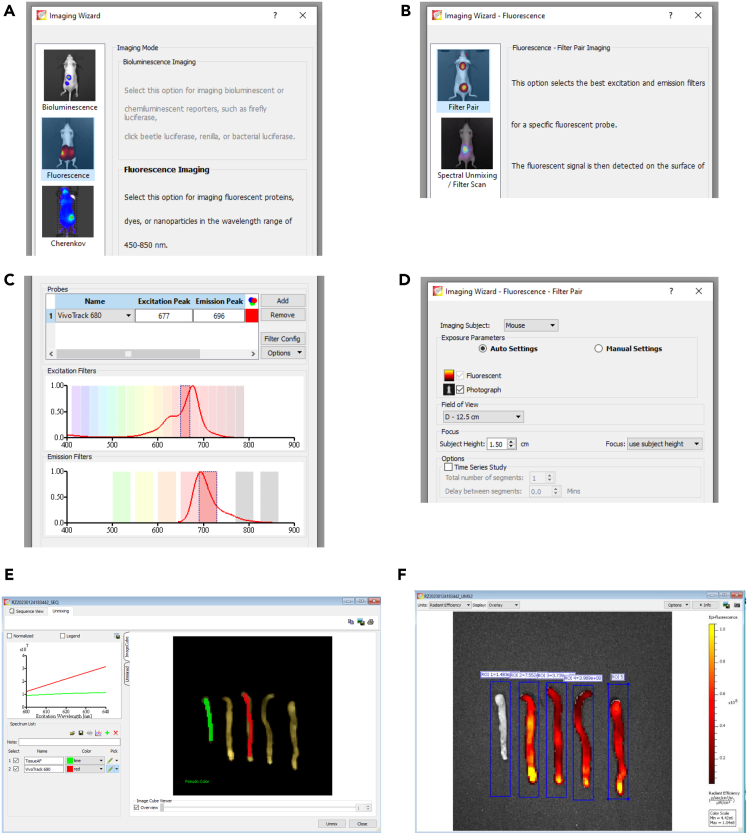
Settings for fluorescence image acquisition using the Living Image software (A and B) (A) “Fluorescence” mode is chosen for image acquisition and images can either be acquired using (B) filter pair or filter scan mode. (C) The filters are shown in the next window of the software; filters depend on excitation and emission peaks and whether filter pair or filter scan mode is chosen. The representative image shows filter pair mode. (D) The field of view and subject height needs to be adjusted according to the organ used for imaging. Whole mouse body images are usually acquired using a subject height of 1.5 cm. (E) If using filter scan mode, spectral unmixing needs to be performed. Therefore, tissue with autofluorescence and tissue with autofluorescence plus target fluorescence are defined. Spectral unmixing is done automatically by the software. (F) For image analysis, regions of interest (ROI) need to be defined.

This step describes the *ex vivo* imaging of isolated organs and tumor tissue of mice transplanted with VivoTrack680-stained ECP treated splenocytes (Figure 2).

9. Open the Living Image Software and initialize the Lumina III In Vivo Imaging System.
10. Euthanize mice (CD45.2) by cervical dislocation or a method approved by the local animal ethics committee.
11. Excise tumor tissue and colon; place tissues in ice-cold 1X PBS.
  a. Remove residual fat and Peyer’s patches from colon tissue; clear intestine of feces with 1X PBS.
12. Take fluorescence images of tissue samples.
  a. Tap colon samples on a paper tissue to remove excess PBS.
  b. Place colon samples on black surface in the Lumina III.
  c. Take a photograph to ensure positioning of the samples in the center of the image.
  d. Adjust imaging settings for VivoTrack680 dye and capture fluorescence image with auto imaging time:
    i. Excitation: 640 nm.
    ii. Emission: 710 nm.
    iii. Exposure time: Auto.
    iv. Binning: Medium.
    v. F/Stop: 2.
    vi. Lamp Level: High.
    vii. Field of view: D.
    viii. Object height: 1.5 cm (default for mice).
  e. Repeat for tumor tissue.

**CRITICAL:** In order to be able to reduce tissue autofluorescence from isolated tissues during image processing, it is crucial to include control animals, which were not transplanted with fluorescently labeled cells. Except for the transplantation of fluorescently labeled splenocytes, the mice should have received the same treatment as the mice transplanted with splenocytes.

***Note:*** Tissue samples should not be imaged in clear plastic dishes or on clear plastic backgrounds, as these materials can cause high autofluorescence. Tissue samples should not be placed in PBS for imaging to reduce fluorescence background.

***Note:*** Any additional tissues of interest can be isolated and analyzed in this step.

### Lamina propria dissociation

**Timing: 1.5 h**

This step describes colon digestion using the Lamina Propria Dissociation Kit (Miltenyi, 130-097-410) to isolate intestinal immune cells according to manufacturer’s instructions.

**Figure 3:**
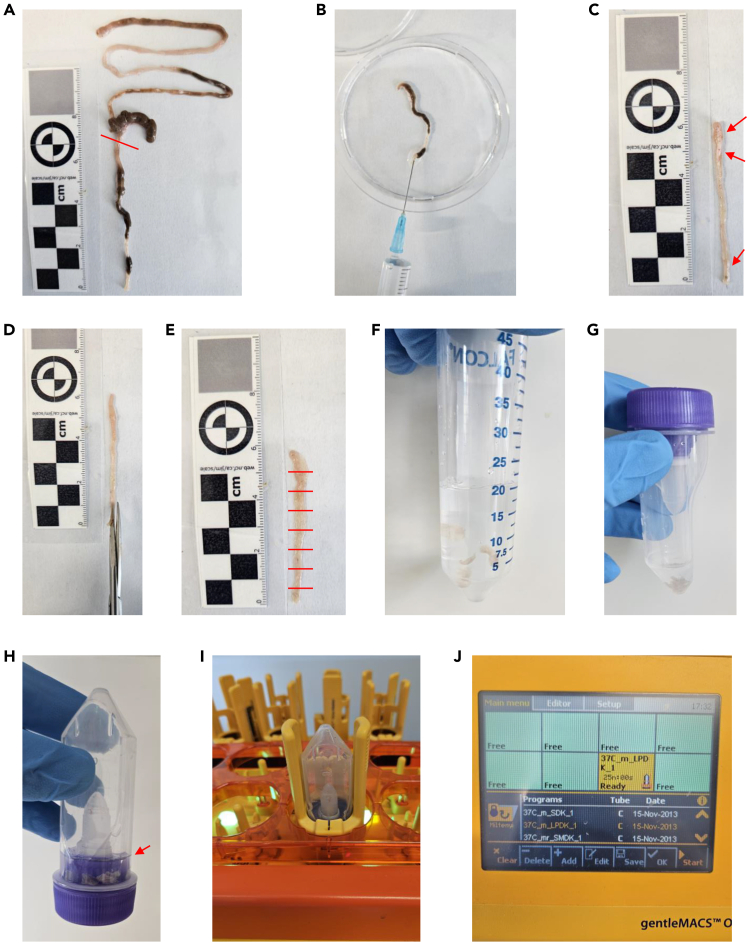
Tissue preparation for lamina propria dissociation (A) Isolate the colon of the mice. To separate the colon from the small intestine, cut the intestinal tissue below the caecum (red line). The lower part is used for colon lamina propria dissociation. (B) Flush the colon with PBS to remove feces. (C) Remove intestinal fat (red arrows) and Peyer’s patches. (D) Cut the colon samples longitudinally as indicated in the picture. (E) Cut the tissue laterally into pieces of ∼0.5 cm length (red lines). (F) Transfer the tissue pieces into 50 mL tubes for predigestion. (G) After predigestion, transfer the tissue into gentleMACS C Tubes. (H) Turn the tubes so that all liquid (digestion mix) and tissue are in the lid of the gentleMACS C Tube (red arrow). Make sure now tissue pieces remain in the bottom of the tube. (I and J) Place the gentleMACS C Tube containing the tissue onto the gentleMACS dissociatior, add the heaters and run the lamina propria dissociation protocol.
15. Transfer tissue pieces into a 50 mL tube containing 20 mL predigestion mix.
16. Incubate the sample for 20 min at 37°C and 300 rpm.
17. Vortex for 10 s.
18. Transfer residual tissue pieces in a new 50 mL tube containing 20 mL predigestion mix.
19. Incubate the sample for 20 min at 37°C and 300 rpm.
20. Vortex for 10 s.
21. Transfer residual tissue pieces in a new 50 mL tube containing 20 mL HBSS (w/o Ca^2+^ and Mg^2+^), supplemented with 10 mM HEPES.
22. Incubate the sample for 20 min at 37°C and 300 rpm.
23. Vortex for 10 s.
24. Prepare enzyme mix in a gentleMACS C Tube.
  a. 2.35 mL pre-warmed digestion solution.
  b. 100 μL Enzyme D.
  c. 50 μL Enzyme R.
  d. 12.5 μL Enzyme A.
25. Transfer tissue pieces into the gentleMACS C Tube.
26. Run the following program on the gentleMACS dissociator with heater: 37C_m_LPDK_1.
27. Spin the gentleMACS tube for 30 s at 300 × *g.*
28. Resuspend sample, add 5 mL of 1X PBS and apply cell suspension to a 100 μm strainer placed on a 50 mL tube.
29. Wash strainer with 10 mL 1X PBS.
30. Centrifuge at 300 × *g* for 10 min at 4°C.
31. Aspirate supernatant completely.
32. Resuspend cells in 1X PBS and proceed to FACS staining.

***Note:*** Cells in the supernatants of the predigestion medium can be collected if epithelial cells should be analyzed. This protocol aimed to analyze immune cells in the lamina propria only and epithelial cells were discarded.

***Note:*** The digestion can also be performed using a gentleMACS dissociator without heaters; see manufacturer’s instructions for details.

13. Weigh intestinal samples; weight is needed to calculate total number of cells per mg tissue.
14. Cut the intestine longitudinally and then laterally into pieces of ∼0.5 cm length (Figure 3).

### Tumor digestion and lymphocyte isolation

**Timing: 2 h**

This step describes the digestion of B16.F10 tumor tissue in order to analyze infiltration of immune cells. Lymphocytes are enriched by Percoll density centrifugation.

**Figure 4:**
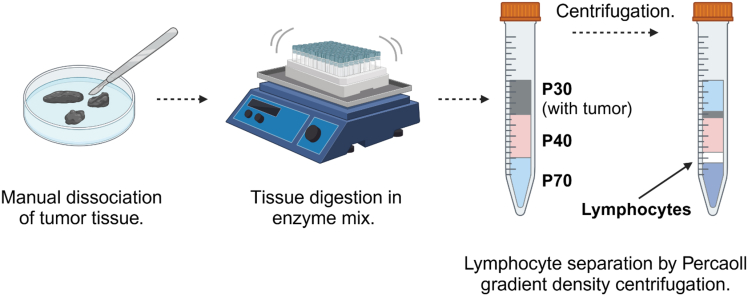
Percoll density centrifugation used for isolation of tumor-infiltrating lymphocytes Tumor tissue is digested by manual dissociation and incubated in tissue digestion medium. Subsequently, dissociated tissue is resuspended in P30 Percoll and layered over P70 and P40 Percoll. After centrifugation, lymphocytes separate above the P70 phase.
39. Centrifuge at 680 × *g* for 20 min at 20°C–25°C; acceleration at 2, no brake.
40. Aspirate and discard the top phase and the first half of the second phase; carefully collect the lymphocytes above the bottom phase and transfer into a 50 mL tube.
  a. Add 25 mL 1X PBS and spin down at 500 × *g* for 10 min at 4°C.
  b. Carefully aspirate and discard the supernatant.
  c. Resuspend cells in PBS and proceed to FACS staining.

***Note:*** The tumor digestion protocol should be adapted if other tumor tissue than B16.F10 is processed.

33. Weigh tumor samples; weight is needed to calculate total number of cells per mg tissue.
34. For each tumor, add 5 mL warm (37°C) tumor digestion medium in a 15 mL tube.
35. Chop the tumor into small pieces using scissors or a scalpel and transfer into the digestion medium.
36. Digest the tumor tissue at 37°C with constant shaking at 200–300 rpm.
37. Pass the digested tumor through a 100 μm strainer into a 50 mL tube, wash filter with 25 mL 1X PBS.
  a. Spin down cells at 400 × *g* for 10 min at 4°C.
  b. Carefully aspirate and discard the supernatant.
  c. Resuspend cell pellet in 3 mL P30.
38. Layer the Percoll gradient in a 15 mL tube (see Figure 4).
  a. Add 3 mL P70.
  b. Carefully layer with 4 mL P40.
  c. Carefully layer with 3 mL P30 containing digested tumor tissue.

***Alternative:*** The tumor tissue can also be digested with commercially available products (e.g. the Tumor Dissociation Kit, mouse, Miltenyi #130-096-730 in combination with a gentleMACS Octo Dissociator with heater).

**CRITICAL:** The tumors need to be cut into small pieces for efficient digestion. Bigger tissue fragments can cause incomplete digestion and lower numbers of lymphocytes for analysis.

**CRITICAL:** Percoll is toxic to the cells and digested tumors should be kept in Percoll as short as possible to enhance lymphocyte viability.

### FACS-based analysis of organ-specific cell migration

**Timing: 3 h**

This part of the protocol describes the analysis of recipient and donor immune cells by flow cytometry. This analysis is important to quantify the migration of fluorescently labeled donor splenocytes into analyzed target organs.

41. Distribute dissociated lamina propria samples and lymphocytes isolated from tumors equally into 96-well round bottom plates for FACS staining.
  a. Spin down cells at 500 × *g* for 3 min at 4°C.
  b. Discard supernatant.
42. Resuspend samples in 100 μL viability staining.
  a. LIVE/DEAD Fixable Aqua Dead Cell Stain Kit, 1:1000 in 1X PBS.
  b. Incubate 15 min at 4°C.
43. Wash cells twice with 200 µL flow cytometry staining buffer (spin cells at 500 × *g* for 3 min at 4°C).
44. Resuspend samples in 50 μL of Fc receptor blocking.
  a. Anti-mouse CD16/CD32, 1:25 in flow cytometry staining buffer.
  b. Incubate 10 min at 4°C.
45. Directly add 50 μL of surface stain antibody mix (total volume is 100 μL).
  a. Panel 1: anti-mouse CD45.1 (FITC), anti-mouse CD45.2 (Pacific Blue); both 1:50 in flow cytometry staining buffer (final dilution is 1:100).
  b. Panel 2: anti-mouse CD45.2 (PE), anti-mouse CD11b (FITC); dilute 1:50 and 1:10 in flow cytometry staining buffer, respectively (final dilution is 1:100 and 1:20).
  c. Incubate 30 min at 4°C.
46. Wash cells twice with 200 µL flow cytometry staining buffer (spin cells at 500 × *g* for 3 min at 4°C).
47. Panel 1: resuspend cells in 150 μL flow cytometry staining buffer and keep at 4°C in the dark until acquisition.

**CRITICAL:** Panel 1 is not fixed. Acquire Panel 1 within 1 h after staining.

48. Panel 2: Resuspend cells in 200 μL fixation/permeabilization solution (BD Cytofix/Cytoperm).
  a. Incubate 40 min at 4°C.
  b. Spin cells down at 750 × *g* for 3 min at 4 °C.
  c. Wash cells twice with 200 μL 1X BD Perm/Wash Buffer; spin at 750 × *g* for 3 min at 4°C.
  d. Resuspend cells in 100 μL intracellular stain.
    i. Anti-mouse/human Arginase 1 (eFluor450); 1:50 in 1X BD Perm/Wash.
    ii. Incubate 30 min at 4°C.
    iii. Wash cells twice with 200 μL of 1X BD Perm/Wash Buffer.
    iv. Resuspend cells in 150 μL of 1X BD Perm/Wash Buffer.
49. Before acquisition, add 10 μL counting beads into each sample; vortex well before acquisition.
50. Analyze samples using a flow cytometer.

**Pause Point:** Panel 2 is fixed and can be kept at 4°C for up to 24 h before acquisition.

***Note:*** The viability stain and the fluorophores reported in this protocol are just suggestions and can be changed according to the availability of antibodies or machines used for analysis. However, it is recommended to spare analysis channels for APC or APC-Cy7 due to high fluorescence overlap with the VivoTrack680 dye.

***Note:*** The red laser and channel for Alexa Fluor 700 was suitable for VivoTrack680 FACS analysis in our settings, but this might need to be adjusted depending on the available analysis channels and FACS devices (excitation maximum is at 676 nm; emission maximum is at 696 nm).

### Data analysis

**Timing: 1–2 h**

This part of the protocol describes the analysis of the primary data collected by *ex vivo* imaging and flow cytometry.

51. Analysis of *ex vivo* imaging data.
  a. Analyze fluorescence images using the Living Image software.
  b. Import images into analysis software.
  c. Perform spectral unmixing.
    **CRITICAL:** Define tissue from mice without splenocyte transfer as autofluorescence control; define tissue from mice with splenocyte transfer as autofluorescence plus fluorescence sample.
  d. Define the region of interest (ROI) and measure fluorescence signals in ROI as “Avg Radiant Efficiency [p/s/cm^2^/sr]/[μW/cm^2^]”.
  e. For representative images, adjust the color scale. Control tissue without fluorescence should not show any color staining.
52. Analysis of flow cytometry data.
  a. Export acquired flow data as fcs files and open in the FlowJo analysis software.
    i. Gate on all cells and include a separate gate for the counting beads.
    ii. Gate on single cells.
    iii. Gate on viable cells.
    iv. Gate on CD45.2^+^ recipient immune cells.
      ***Optional:*** A gate for CD45.1^+^ cells can be included; these can be excluded from the analysis.
    v. Gate on VivoTrack680^+^ cells.
    vi. Export “counts” of counting beads and CD45.2^+^ VivoTrack680^+^ cells in table format.
  b. Calculate total number of cells per sample according to manufacturer’s instructions (e.g. 123count eBeads Counting Beads). This calculation is dependent on the exact counting beads used for analysis.
  c. Calculate total cells per mg tissue, based on total tissue weight before digestion.
    **Pause Point:** Researchers can pause the protocol after data acquisition and analysis can be performed at a later time.
    ***Note:*** We have used the FlowJo software to analyze the flow cytometry data. If not available, any other analysis software for flow cytometry data can be used instead.
    **CRITICAL:** Note sample resuspension volumes, volume of beads, concentration of beads (usually LOT-specific) and tissue weights to be able to calculate total cell counts after flow cytometry analysis.

## Expected outcomes

ECP was shown to be highly effective to control ICI-induced colitis without negatively affecting anti-tumor immunity. We had hypothesized that ECP-treated splenocytes are primarily migrating into inflamed intestinal tissue, whereas they spare the tumor microenvironment. In the target organs, we hypothesized that these ECP-treated splenocytes are phagocytosed by local antigen presenting cells (APCs). Although ECP was found effective in clinical practice for the treatment of immune-related adverse events (irAEs) and Graft-versus-Host Disease (GvHD), its mechanism how to induce local immunosuppression remained unclear.^2^^,^^3^^,^^4^^,^^5^ Using fluorescence labeling of donor splenocytes, we attempted to understand if ECP-treated splenocytes migrate into inflamed organs or tumor tissue. We next investigated whether phagocytes in the respective tissues clear the ECP-treated splenocytes. Co-cultures of ECP-treated splenocytes and macrophages *in vitro* showed that APCs were rendered immunosuppressive upon phagocytosis of ECP-treated splenocytes. We aimed to determine whether the phagocytic uptake of ECP-treated cells also polarizes macrophages towards an immunosuppressive phenotype *in vivo*.

Using our fluorescence labeling approach described in this protocol, we could trace the infiltration of ECP-treated splenocytes into the inflamed intestinal tract, but not into the tumor microenvironment.^1^ The infiltration analysis can be extended for any organ, and different fluorescent dyes could be combined to investigate the tissue-specific migration dependent on the treatment of the splenocytes. The use of CD45.1 donor animals and CD45.2 recipient animals enabled us to specifically analyze immune cells of recipient and donor animals. Based on our aims, we decided to investigate colon and tumor samples, but other organs could be dissociated to understand immune cell infiltration.

We demonstrated that the inflamed colitis tissue is highly infiltrated by ECP-treated splenocytes, whereas the tumor tissue shows minimal infiltration. This was illustrated by *ex vivo* fluorescence imaging. The data was verified by flow cytometry after dissociation of colitis and tumor tissues.^1^

Collectively, we present a protocol to track splenocytes after labeling with a fluorescence dye. The model can be adapted to track other cell types, or cells labeled with a different fluorescent dye. Further, this model can be adapted to track cells in other disease models, or be used in models with other tumor types.

## Limitations

This protocol was adapted to meet the specific need to track ECP-treated splenocytes in tumor-bearing mice with ICI-induced colitis. This is a very unique and complex disease setting, but the method can be adapted according to specific scientific needs.

We have developed and tested this protocol using the VivoTrack680 fluorescent dye, but Revvity has more dyes in their portfolio to be used for *in vivo* imaging. However, fluorophores in the far-red spectrum are known to have the strongest tissue penetrance and are therefore recommended to be used for *in vivo* and *ex vivo* imaging, especially if low fluorescence signals are expected for some tissues.

The fluorescence imaging was developed using the Lumina III imaging system. If users have no access to this device, other animal imaging devices can be used, or *ex vivo* organ imaging can be skipped and the infiltration of splenocytes into different organs can be analyzed by flow cytometry only.

The protocol for tumor dissociation was adapted for B16.F10 tumors, which are generally soft tumors. If researchers are using other tumor cell lines, the tumor digestion step needs to be adapted. Tumor dissociation kits are also commercially available with enzyme recommendations depending on the tumor cell lines (e.g. Tumor Dissociation Kit, Miltenyi). Suitable protocols can be found online.^6^

In order to achieve good tissue dissociation, we were using the Lamina Propria Dissociation Kit and the related gentleMACS Octo Dissociator with heater (all Miltenyi). These might not be available to all labs, and researchers can also dissociate intestinal samples using collagenase D and DNase I.^7^^,^^8^

The schedule to induce colitis with DSS and ICI was adapted to our animal facility. Researchers should be aware that the colitis severity might be different depending on mouse strains, gender, and age of the mice and hygiene status of the facility.^9^

## Troubleshooting

### Problem 1

Larger tissue fragments remain in lamina propria samples after lamina propria dissociation.

### Potential solution

Insufficient tissue dissociation can have multiple reasons. It is crucial to remove Peyer’s patches and intestinal fat from the samples before starting the dissociation. Ensure that all tissue and enzyme mix are positioned in the cap of the gentleMACS tube to facilitate proper tissue dissociation.

### Problem 2

Large tissue fragments remain after digestion of tumor samples.

### Potential solution

For the tumor digestion step, it is crucial to remove all skin and adhesive tissues from the isolated tumors. B16.F10 tumors are generally soft and easy to digest. Make sure that tumor tissue is cut into small pieces before transferring into the digestion solution. Digestion medium and digestion times might need variation if used for other tumors than B16.F10. After digestion, remaining parts can be mashed through a 100 μm cell strainer to enhance tissue dissociation.

### Problem 3

There is no apparent lymphocyte layer after Percoll gradient centrifugation.

### Potential solution

When layering Percoll solutions, carefully layer each phase without disturbing the interphase between different concentrations. All Percoll solutions should be at 20°C–25°C (RT) before use. The centrifugation step needs to be done at 20°C–25°C (RT). Keeping Percoll on ice or centrifugation below 20°C causes changes in density and will interfere with sample separation efficacy.

### Problem 4

Low fluorescence intensity is observed for *ex vivo* imaging of isolated organs.

### Potential solution

Fluorescence intensity is highly dependent on the number of tissue-infiltrating cells. If low cell counts are expected, the number of transplanted cells can be increased. Additionally, camera settings (exposure time, binning and F/Stop) can be adjusted to enhance sensitivity. However, it is recommended to take an image with “auto exposure” first to balance fluorescence signals and background noise. Using VivoTrack680 is recommended as it has a good tissue penetrance, but other fluorescent dyes could be evaluated if needed.

### Problem 5

High fluorescence background and autofluorescence are observed during *ex vivo* imaging of isolated organs.

### Potential solution

It is recommended to apply a chlorophyll-free diet to the mice for about 2 weeks before organ imaging, as chlorophyll causes high autofluorescence. This is especially important if images of the gastrointestinal tract are taken. The cell lines used for tumor induction should not express any fluorescent reporter to minimize fluorescence overlap.

### Problem 6

Mice show insufficient tumor growth and insufficient colitis development.

### Potential solution

The kinetics of tumor growth is highly dependent on the cell line used for tumor induction, genetic modifications of the cell lines, number of injected cells and the genetic background of mice used as recipients. It is recommended to keep the cell lines in a logarithmic growth phase before injection for successful tumor growth. Changing cell lines or treatments might need an adaption of the protocol to facilitate sufficient tumor growth. The development of DSS-mediated colitis is highly dependent on the recipient mouse strain, the gender of the mice, the age and the microbiome. Small pilot experiments might be recommended to evaluate a DSS dosage, which is sufficient to induce colitis.

### Problem 7

No positive staining for CD45.1, CD45.2, CD11b or Arginase 1.

### Potential solution

All targets used for staining in this protocol are generally easy to stain with commercially available antibody clones. If surface markers are found negative, please verify that the antibody was added into the staining mix and that the antibody is working. CD45.1 is usually not found, or only in low numbers, in this model, as these cells undergo apoptosis after ECP therapy and are phagocytosed after transplantation. To verify that the antibodies are working, splenocytes from CD45.1 and CD45.2 mice can be stained as positive controls. For Arginase 1 staining, permeabilization of the cells is critical. The products and timing described in this protocol are sufficient for Arginase 1 staining. Substitutions with other products and changes of the protocol should be critically evaluated. VivoTrack680 exhibits strong fluorescence; make sure that this color can be acquired and compensated with the flow cytometer used for analysis.

## Resource availability

### Lead contact

Further information and requests for resources and reagents should be directed to and will be fulfilled by the lead contact, Robert Zeiser (robert.zeiser@uniklinik-freiburg.de).

### Technical contact

Questions regarding the technical specifics of performing the protocol should be directed to the technical contact, Lukas M. Braun (lukas.braun@uniklinik-freiburg.de).

### Materials availability

This study did not generate new unique reagents.

### Data and code availability

This study did not generate any unique datasets or codes.

## Acknowledgments

Parts of the figures and the graphical abstract were created using BioRender.com. This study was supported by grants from the Deutsche Forschungsgemeinschaft (DFG, German Research Foundation): Project-ID 441891347—SFB1479, Project-ID 259373024—TRR167, and Project-ID 256073931—CRC1160; the European Union: EU proposal no. ERC-2022-ADG, Project 101094168—AlloCure (ERC Advanced grant); EU project: Project-ID 101119855—exTra; ERANET Transcan—PIXEL; ERA-NET Transcan—SmartCART; the CIBSS—EXC-2189—Project-ID 390939984; the Deutsche Krebshilfe (70114655); the Jose-Carreras Leukemia foundation (DJCLS 09R/2022); the Leukemia and Lymphoma Society (7030-23); and the German Cancer Consortium (DKTK) (all to R.Z.).

We thank the Lighthouse Core Facility at the University Medical Center Freiburg. The Lighthouse Core Facility is funded in part by the Medical Faculty, University of Freiburg (project nos. 2023/A2-Fol; 2021/B3-Fol), the DKTK, the Mertelsmann Foundation, and the DFG (project no. 450392965). We thank the staff of the animal facility at the University Medical Center Freiburg for their support.

## Author contributions

Conceptualization, L.M.B. and R.Z.; methodology, L.M.B. and R.Z.; investigation, L.M.B.; formal analysis, L.M.B.; writing – original draft, L.M.B. and R.Z.; writing – review and editing, L.M.B. and R.Z.; funding acquisition, R.Z.; supervision, R.Z.

## Declaration of interests

R.Z. has received honoraria from Novartis, Incyte, Sanofi, Neovii, and Mallinckrodt. All honoraria and support were outside this work.

## References

[1] Braun L.M., Giesler S., Andrieux G., Riemer R., Talvard-Balland N., Duquesne S., Rückert T., Unger S., Kreutmair S., Zwick M. (2025). Adiponectin reduces immune checkpoint inhibitor-induced inflammation without blocking anti-tumor immunity. Cancer Cell.

[2] Apostolova P., Unger S., von Bubnoff D., Meiss F., Becher B., Zeiser R. (2020). Extracorporeal Photopheresis for Colitis Induced by Checkpoint-Inhibitor Therapy. N. Engl. J. Med..

[3] Ertl C., Ruf T., Hammann L., Piseddu I., Wang Y., Schmitt C., Garza Vazquez X., Kabakci C., Bonczkowitz P., de Toni E.N. (2024). Extracorporeal photopheresis vs. systemic immunosuppression for immune-related adverse events: Interim analysis of a prospective two-arm study. Eur. J. Cancer.

[4] Ruf T., Rahimi F., Anz D., Tufman A., Salzer S., Zierold S., Tomsitz D., French L.E., Heinzerling L. (2024). Extracorporeal Photopheresis as a Treatment Option for Immune-Related Adverse Events: Two Case Reports and a Prospective Study. J. Immunother..

[5] Maas-Bauer K., Kiote-Schmidt C., Bertz H., Apostolova P., Wäsch R., Ihorst G., Finke J., Zeiser R. (2021). Ruxolitinib-ECP combination treatment for refractory severe chronic graft-versus-host disease. Bone Marrow Transplant..

[6] Xiao L., Yi Q. (2023). Isolation of adoptively transferred CD8(+) T cells in mouse tumor tissues for lipid peroxidation detection. STAR Protoc..

[7] Hulsdunker J., Zeiser R. (2016). In Vivo Myeloperoxidase Imaging and Flow Cytometry Analysis of Intestinal Myeloid Cells. Methods Mol. Biol..

[8] Hulsdunker J., Ottmuller K.J., Neeff H.P., Koyama M., Gao Z., Thomas O.S., Follo M., Al-Ahmad A., Prinz G., Duquesne S. (2018). Neutrophils provide cellular communication between ileum and mesenteric lymph nodes at graft-versus-host disease onset. Blood.

[9] Nicolas A.M., Pesic M., Rödel F., Fokas E., Greten F.R. (2022). Image-guided radiotherapy in an orthotopic mouse model of rectal cancer. STAR Protoc..

